# Dr. Thompson's Case of Abscess of the Liver

**Published:** 1812-04

**Authors:** D. Thompson

**Affiliations:** Whitby


*To the Editors of the Medical and Physical Journal.*

GENTLEMEN,

ABOUT three years since, a laboring man, aged 50, came to me for advice with the following symptoms. A dull pain, or rather sense of uneasiness, in the region of the liver, attended with short dry cough, alternations of heat and cold, quick soft pulse, diarrhœa, and much emaciation; but the symptoms of which he most complained were constant nausea, and, as he termed it, a stinking taste in his mouth. He also informed me that, to use his own expression, he sometimes threw up a great gush of corruption. He had been declining in health, with disordered stomach, and some, though not severe, pains of the side for many months; but the vomiting, and putrid taste in his mouth, had only continued a few weeks. I examined the hypochondriac region very accurately, but could discover no symptom of disease except a slight increase of pain upon pressure. The medicines I ordered him were a gentle emetic every second day, and on the intermediate days, and on the same days after vomiting, an infusion of bark, with sulphuric acid and opium. I desired him to refrain from strong liquors, to which he had been much addicted; to take light nourishing nourishing food, with brisk bottled ale, or porter; and, as often as he should find himself able, to ride out either on horseback or in a cart. After about three weeks I saw him again, much improved in his looks, and with every symptom of disease abated. The sickness and foul taste in his mouth had nearly left him; his diarrhœa had ceased; he was less feverish; and his appetite was returning. He informed me, that, upon first taking the emetics, he had vomited a large quantity of very fetid matter; that the quantity had gradually diminished, and that latterly none had appeared. I advised him to continue his bark infusion, but to take an emetic only as often as the nausea, and bad taste in his mouth, should return. About three months afterwards I saw him again, perfectly free from complaint. He had returned to his work, and declared that he was as well as ever he had been in his life. I have not seen him since.

From the symptoms above described, I concluded that an adhesive inflammation had taken place between the upper surface of the liver and stomach, and that an abscess, having formed in the former, had burst into the latter. From this view of the case, though I certainly considered the man in much danger, the treatment proper to be pursued was simple and obvious. It consisted merely in giving free vent to the matter, in correcting putrescency, lessening irritation, and supporting the strength. A doubt will perhaps arise in the minds of some of your readers, whether the matter might not have been discharged as effectually, and perhaps with less danger, by brisk cathartics. The reflection did not escape me at the time, but I preferred emetics because I thought they would effect that purpose quicker, and with more certainty, and because I considered that the action of vomiting was likely to empty the abscess more completely than could be done by any other means. To assist in this object I also recommended jolting either on horseback or in a cart.

I should not have thought of troubling you with the above case, but for the resemblance which it bears to that related by Dr. Paxton; for, however much they have differed in other respects, in their nature they were essentially the same, and I therefore thought it might be of some use to have them recorded in the same volume. There was one circumstance, indeed, common to both, very deserving of attention, and which I think cannot be pointed out too clearly—in neither of them was the pain of the side at any time severe. The liver is certainly an organ possessing less sensibility than most others, and hence its diseases, being frequently unmarked by any very striking symptoms, may easily escape escape detection, or, if detected, may appear too trifling to demand that active practice which ought to be adopted. That this happened in Dr. Paxton's case, I infer as well from the manner in which it was treated, as from that gentleman's candid confession, in as much as his own judgment was concerned. I fear indeed it happens too often.

Hepatic complaints are, I think, more obscure, and more frequently mistaken than those of any other organ in the human body. I am tired of that sweeping and ill-defined term bilious, and should be grateful to any persons, possessing the means of extensive observation, and of correcting his opinions by dissection, who would present us with something like a systematic arrangement of the diseases of the liver, and with the symptoms by which they may be known and distinguished; for, notwithstanding we have several good works connected with this subject, from that of Dr. Saunders, downwards, yet additional, and particularly more systematic, information is certainly much needed.

It has often occurred to me, that, in examining the functions of the hepatic system, we have taken too partial a view, and have been too easily satisfied with first appearances. The magnitude of the liver; the large quantity of venous blood, chiefly from the intestines, and probably tainted and vitiated by their contents, which passes through it; the singular office of the vena portarum; the highly alkalescent quality of the bile; the circumstance of its being discharged into the intestines rather than into the stomach; and more especially the great disproportion between its apparent uses, and the vast preparation made to procure it; have all led me to a suspicion *that the principal purpose of the liver is not the secretion of bile for the mere reasons which we have imagined, but that it ought rather to be considered of the nature of the kidneys, as an excretory rather than a secretory organ, as intended, in fact, rather to remove something noxious from the constitution, than to provide any thing useful for it.*

That the bile, however, does answer some useful purposes, may be concluded from its entering the intestinal canal at its origin, rather than at its termination, for nothing in our wonderful structure has been arranged without design. There can be no doubt of its acting as a stimulus to the intestines, rousing them to action, and enabling them to expel their contents, and by mixing with the chyle it probably blends the oleaginous and aqueous particles more intimately together, and produces other chemical changes unknown to us; but this double purpose of the hepatic system perfectly accords with the wisdom and simplicity every-where observed served in the works of Providence, and instances of similar contrivance may be remarked throughout nature almost innumerable.

If the above conjecture of the functions of the liver be correct, we cannot wonder at the various and anomalous appearances which its derangement exhibits. If its action be excessive, too much,—if deficient, too little,—may be carried out of the system; and, in either case, the blood may contain an undue proportion of those elements proper for digestion, secretion, and the other vital functions, the same as appears to happen in diabetes and ischuria, and the whole system may become disordered. These, I confess, are mere hypothetical conjectures, but they may possibly have the effect of drawing the attention of some of your readers to an organ, and to a class of diseases, which stand in much need of further elucidation. I am, Gentlemen,

Your's, &c.

D. THOMPSON, M.D.

*Whitby, Feb.* 11, 1812.

